# The Influence of Quadruplex Structure in Proximity to P53 Target Sequences on the Transactivation Potential of P53 Alpha Isoforms

**DOI:** 10.3390/ijms21010127

**Published:** 2019-12-24

**Authors:** Otília Porubiaková, Natália Bohálová, Alberto Inga, Natália Vadovičová, Jan Coufal, Miroslav Fojta, Václav Brázda

**Affiliations:** 1 Faculty of Chemistry, Brno University of Technology, Purkyňova 118, 61200 Brno, Czech Republic; o.porubiakova@gmail.com; 2 Institute of Biophysics, Academy of Sciences of the Czech Republic, Královopolská 135, 61265 Brno, Czech Republic; nataliabohalova@gmail.com (N.B.); vadovicovan@mail.muni.cz (N.V.); jac@ibp.cz (J.C.); fojta@ibp.cz (M.F.); 3 Department of Experimental Biology, Faculty of Science, Masaryk University, Kamenice 5, 62500 Brno, Czech Republic; 4 Laboratory of Transcriptional Networks, Department CIBIO, University of Trento, via Sommarive 9, 38123 Trento, Italy; alberto.inga@unitn.it; 5 Department of Biology, Faculty of Medicine, Masaryk University, Kamenice 5, 62500 Brno, Czech Republic

**Keywords:** p53 protein, protein-DNA interaction, transactivation potential

## Abstract

p53 is one of the most studied tumor suppressor proteins that plays an important role in basic biological processes including cell cycle, DNA damage response, apoptosis, and senescence. The human *TP53* gene contains alternative promoters that produce N-terminally truncated proteins and can produce several isoforms due to alternative splicing. p53 function is realized by binding to a specific DNA response element (RE), resulting in the transactivation of target genes. Here, we evaluated the influence of quadruplex DNA structure on the transactivation potential of full-length and N-terminal truncated p53α isoforms in a panel of *S. cerevisiae* luciferase reporter strains. Our results show that a G-quadruplex prone sequence is not sufficient for transcription activation by p53α isoforms, but the presence of this feature in proximity to a p53 RE leads to a significant reduction of transcriptional activity and changes the dynamics between co-expressed p53α isoforms.

## 1. Introduction

The tumor suppressor protein, p53, is called the “guardian of the genome” due to its crucial role in maintaining genetic stability and inhibiting cancer formation [1,2]. To exert this role, once activated after cell injury, p53 induces a number of cellular processes, resulting in cell repair and survival or in programmed cell death [3,4,5]. The canonical p53 protein, also named p53α, FLp53α, or TAp53alpha (hereafter referred to as FLp53α), was the first identified p53 form [6]. Human FLp53α is 393-amino acids long and has seven functional domains. The N-terminal domain contains two transactivation (TA) domains, which are required to induce a distinct subset of p53-target genes. Other domains are a proline-rich domain (PRD), a DNA-binding domain (DBD), a hinge domain (HD), and a C-terminal domain composed of an oligomerization domain (OD) and a negative regulation domain (α) [7]. The negative regulation domain is rich in lysine and undergoes many posttranslational modifications that regulate FLp53α activity and stability [8]. The DBD contains several conserved cysteines and histidines that coordinate Zn^2+^ or Mg^2+^ ions, which are essential for FLp53α conformation and DNA-binding activity [9]. Different N-terminal isoforms of p53α have been identified due to alternative translation initiation, splicing sites, or alternative promoter usage: Δ40p53α, Δ133p53α, and Δ160p53α lack the 39, 132, and 159 N-terminal amino acids, respectively, compared with FLp53α [10,11]. As a consequence, Δ40p53α lacks one of the two TA domains while the other two isoforms lack both TA and the PR domains, plus part of the conserved cysteine box in the DBD [12]. Based on experiments over the past ten years, it has been shown that p53 isoforms are physiologically active proteins. Misregulation of p53 isoform expression can lead to cancer, premature aging, neurodegenerative diseases, or even embryo malformations [13,14].

p53 is part of an extensive transcriptional network that coordinates the response to intracellular and extracellular stresses or damage [5]. The main function of p53 is provided by its interaction with DNA [15,16,17,18,19]. p53 regulates target gene expression mainly by activation of p53-responsive promoters. The DNA response element (RE) for p53 binding comprises two copies of a 5′- RRRC(A/T)(T/A)GYYY-3′ sequence [15,20,21] accommodating the binding of two p53 dimers to form a p53 tetramer that is considered the functional unit for transcriptional modulation [16]. The domain responsible for sequence-specific DNA binding is the core DBD, even though the OD is critical for tetramer formation and modifications to the C-terminal domain influence binding affinity and specificity [22]. p53-DNA interactions with p53 REs are sensitive to DNA topology and this is a key parameter contributing to p53-DNA affinity and specificity [18,23]. It was demonstrated that p53 also binds to various local DNA structures stabilized by DNA topological stress such as cruciforms [24,25], quadruplex [26], triplex [27], bulged [28], and hemicatenate [29] DNAs.

The unicellular yeast *Saccharomyces cerevisiae* has been previously employed to study the transcriptional activity of many human transcription factors including p53 and its isoforms [30,31,32]. Here, we have engineered yeast reporter strains to study the impact of positioning a G-quadruplex (G4) prone sequence alone or in proximity (upstream or downstream) of a p53 RE on the transactivation induced by FLp53α and the N-terminally truncated isoforms (∆40p53α, ∆133p53α, and ∆160p53α), expressed both individually and in combination.

In particular, we investigated whether G4 prone sequences are capable of inducing p53-dependent transactivation per se, and/or whether they modify transcription when present in close proximity to an established p53 binding site. We also investigated whether G4 prone sequences impact on the crosstalk between co-expressed p53 isoforms and mapped the presence of G4 forming sequences nearby p53 PUMA RE in genomic context. Our results further emphasize the potential role of structural DNA features as modifiers of p53 protein functions at target promoter sites.

## 2. Results

### 2.1. Construction of Isogenic Yeast Strains

**Figure 1:**
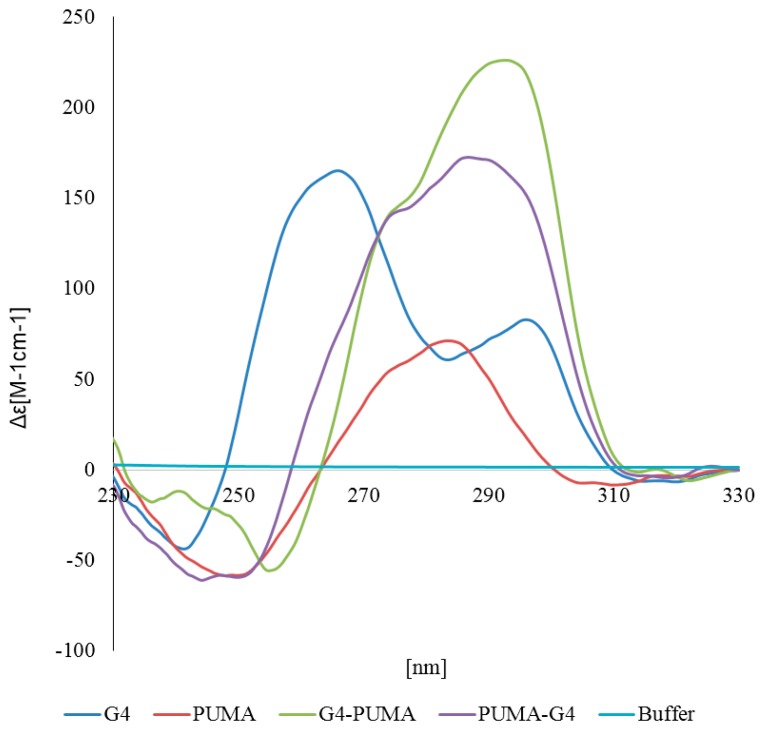
Circular Dichroism }CD] spectroscopy of used DNA sequences. CD spectra of the buffer (light blue), and oligonucleotides from the Table 1 (G4, blue, PUMA-red, G4-PUMA-green, PUMA-G4 violet).

To elucidate the influence of a G4 on p53α transcriptional activity, we exploited yeast isogenic reporters. We used the following G-rich DNA sequence GGGGCGGGGGACGGGGGAGGGG, which is very highly prone to form a G4, based on the propensity score given by the G4Hunter tool [33,34] (G4Hunter score 3.182), which is even higher than the sequence from the c-Myc promoter region (G4Hunter score 2.941) where the presence of the G4 structure has been evaluated both in vitro and in vivo [35,36]. We confirmed the propensity of this sequence to form G4 by CD spectroscopy (Figure 1). The measurements showed that the G-rich sequence forms a hybrid type of G4 with dominant parallel G4 represented by the peak at 264 nm and an antiparallel G4 structure resulting in the secondary peak at 295 nm. The slow drop off of the curve after the typical 264 nm peak is in keeping with the evidence that topologically different G4 intermediates may coexist [37,38]. Sequences with an additional PUMA p53RE region showed higher preference for the antiparallel G4 structure with a more prominent peak around 295 nm.

**Figure 2:**
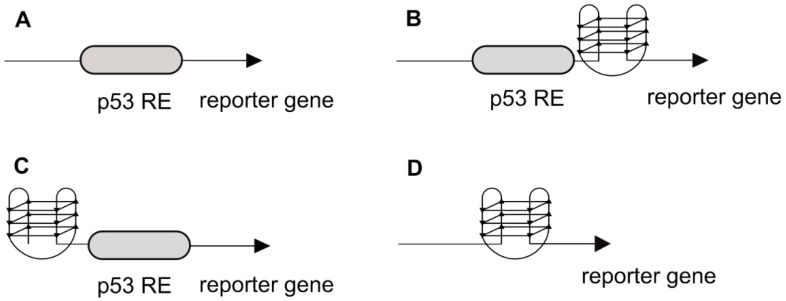
Scheme of the tested sequences in the luciferase reporter promoter region.

Next, we integrated the p53 RE derived from the human PUMA/BBC3 promoter and the G4 sequence alone or combined upstream of a minimal promoter driving the luciferase reporter gene at the *ade2* locus in yeast. Two versions of the combined element were constructed, differing in the position of the G4 sequence either upstream or downstream of the p53 RE (Figure 2).

### 2.2. Transactivation Activity of p53α

**Figure 3:**
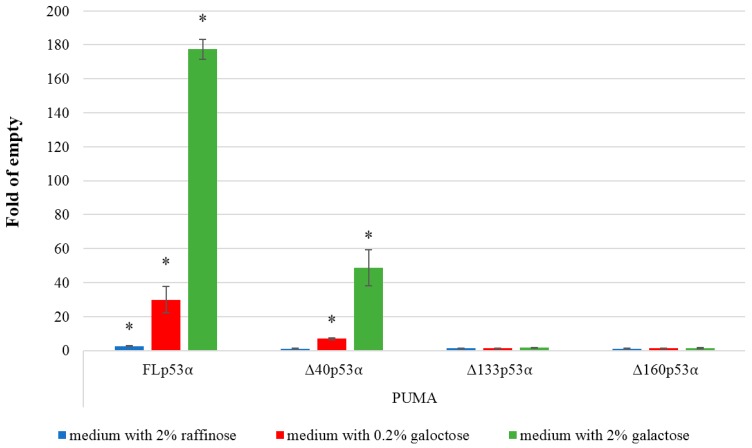
p53-dependent transactivation potential in yeast. All p53α isoforms are expressed under an inducible GAL1 promoter. Histograms show the average fold induction over empty vector in three biological replicates (mean ± S.D.). The results with three levels of p53 induction (no induction, moderate, high) obtained after 24 h in inducing media are presented. Asterisks indicate a significant induction of p53 dependent transactivation (*p* < 0.05).

The reporter yeast strains were used to measure the transactivation potential of four p53α isoforms. First, exploiting the galactose inducible system to control p53 expression, we analyzed the level of transcription of the reporter in the presence of the PUMA p53RE without galactose and with 0.2% or 2% galactose. The results showed that both FLp53α and ∆40p53α transactivate the reporter, although to different extents (Figure 3). Increasing the amount of galactose led to a proportional increase in transactivation for both isoforms. The ∆133α and ∆160α isoforms did not induce transactivation of the PUMA p53 RE.

**Figure 4:**
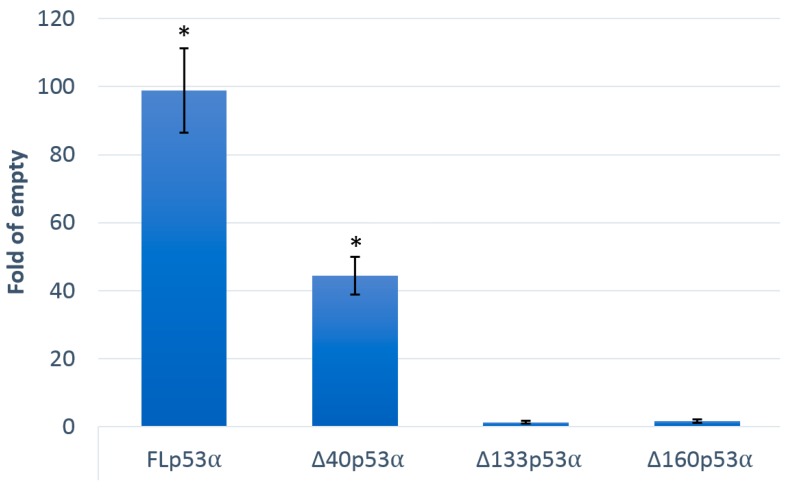
p53-dependent transactivation potential in yeast. All p53α isoforms are expressed under a constitutive glyceraldehyde-3-phosphate dehydrogenase (*GPD*) promoter. The results for the indicated p53α isoforms obtained after 24 h in media without induction are presented. Asterisks indicate a significant induction of p53 dependent transactivation (*p* < 0.05).

Similarly, the transactivation potential of constitutively expressed p53 (GPD promoter) was significantly higher for the FLp53α isoform compared to the ∆40p53α isoform, while ∆133 and ∆160 isoforms were not able to transactivate the reporter (Figure 4).

**Figure 5:**
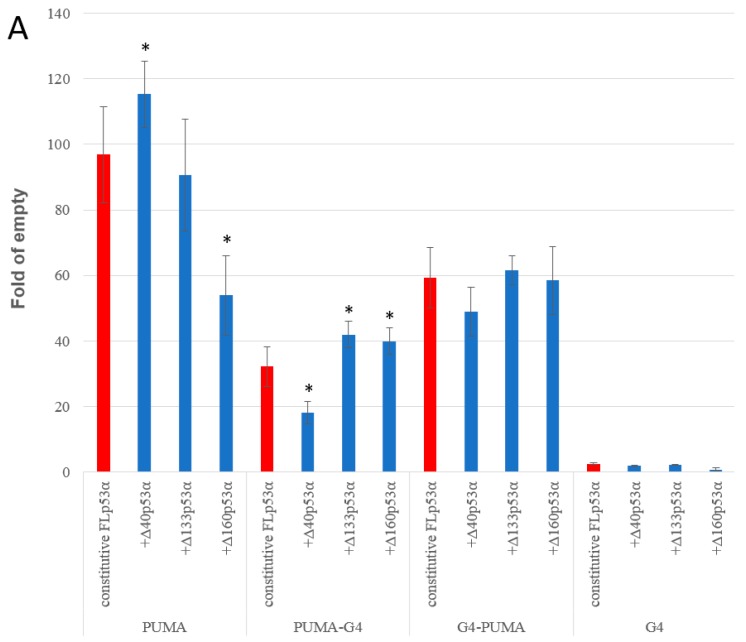

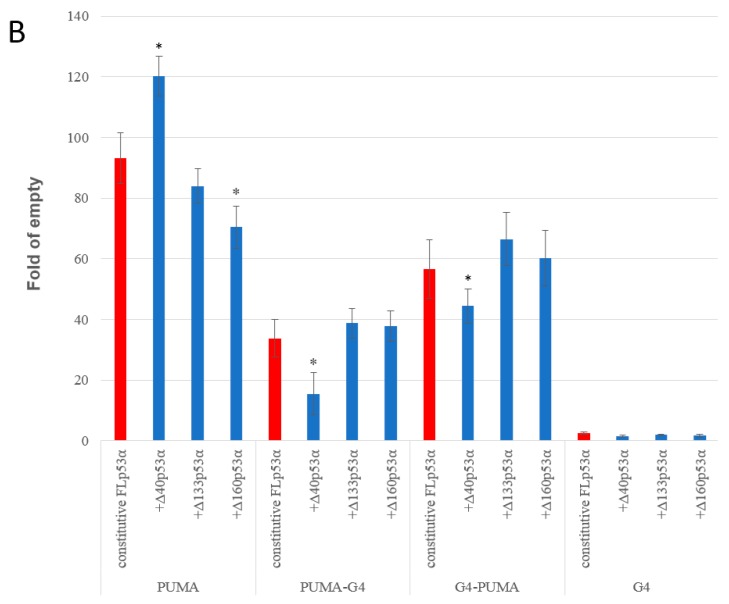
Influence of ∆p53α isoforms with inducible expression on transactivation activity of constitutively expressed FLp53α. (**A**) in media with 0.2 % galactose; (**B**) in media with 2% galactose. Four isogenic yeast strains were used, with the p53 target site (PUMA), with the p53 target site after G4 forming sequence (G4-PUMA), with the p53 target site before G-quadruplex forming sequence (PUMA-G4), and the G4 forming sequence upstream of the luciferase gene. Asterisks indicate a significant induction of p53-dependent transactivation (*p* < 0.05).

**Figure 6:**
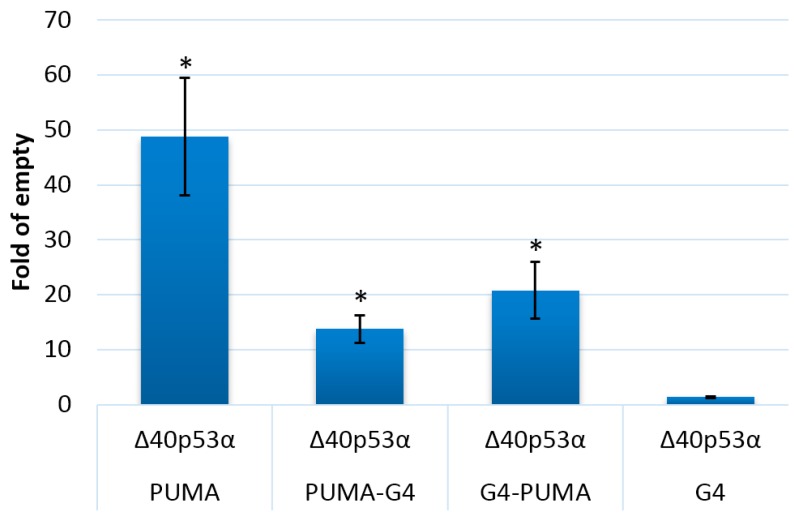
Impact of the G4 prone sequence on Δ40p53α transactivation activity from the PUMA RE. Δ40p53α was expressed under an inducible *GAL1* promoter. Histograms show average fold induction over empty vector in three biological replicates (mean ± S.D.). The results obtained after 24 h in 2% galactose inducing media are presented. Asterisks indicate a significant induction of p53-dependent transactivation (*p* < 0.05).

To elucidate the role of G4 structure on the transcriptional activity p53α isoforms, we tested three additional yeast isogenic strains containing the G4 alone or combinations of the p53 RE with the G4 sequence upstream or downstream. All strains were co-transformed so that the activity of FLp53α expressed alone or combined with the other p53α isoforms could be assessed in the various reporter strains. FLp53α was expressed under the constitutive *GPD* promoter while ∆p53α isoforms were under the *GAL1* promoter and were expressed both at moderate (Figure 5A) and high levels (Figure 5B). Performing western blot of p53 isoforms is challenging due to the lack of commercially available isoform-specific antibodies, but western blot with the DO-1 antibody that detects an N-terminal epitope (residues 11–25) in FLp53α has shown that expression of full-length p53 by the constitutive *GPD* promoter in yeast was not dramatically affected by the co-selection of expression plasmids for p53α isoforms (Figure S1). FLp53α induced transactivation in the strain with just the p53 RE upstream of the luciferase reporter, but had no transactivation activity on G4 alone. The transactivational activity of FLp53α was affected by the G4 sequence placed either upstream or downstream of the p53RE. Interestingly, the presence of the G4 in close proximity to the p53 RE decreased p53-dependent transactivation (Figure 5, red bars), but the position of the G4 sequence influenced this effect. The inhibitory effect was greater with the G4 inserted after the p53 RE (i.e., closer to the TSS) than when the G4 was positioned upstream of the p53 RE. None of the ∆p53α isoforms impacted the low transcription activity of the reporter containing the G4 sequence only. In the p53 PUMA RE reporter strain, ∆160p53α decreased transactivation by FLp53α, particularly when expressed at high levels (Figure 5B). Such a decrease was not observed with ∆40p53α (consistent with the residual transactivation potential of this isoform), but it slightly potentiated FLp53α transactivation activity. However, placing the G4 sequence downstream of the p53 RE led to changes in the apparent functional interaction between co-expressed p53α isoforms, and ∆40p53α gained an inhibitory effect over FLp53α, while ∆133p53α and ∆160p53α lost that property. Indeed, when expressed alone, ∆40p53α was impacted by the presence of the G4 sequence in a manner similar to FLp53α (Figure 6).

## 3. Discussion

**Figure 7:**
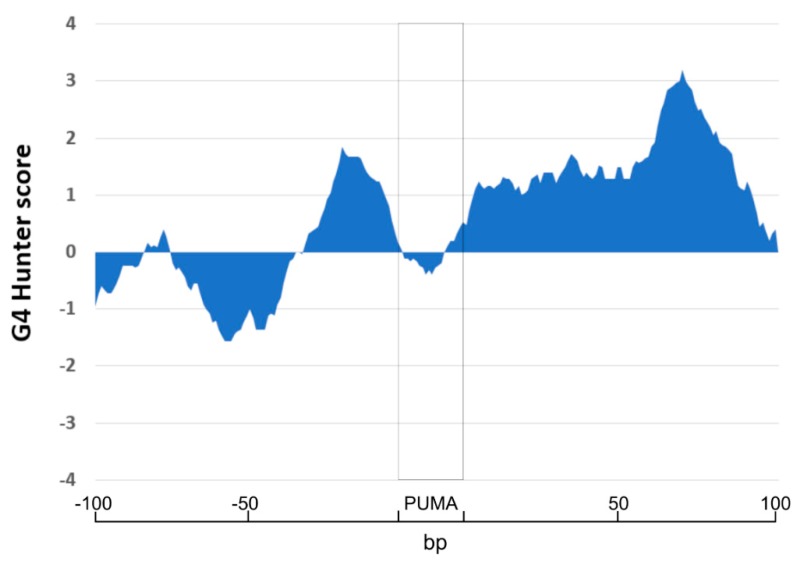
Localization of G4-prone sequences around p53 PUMA RE sequence (100 bp before and after p53 PUMA RE). The analysis of G4-prone sequences has shown that p53 PUMA RE (rectangle) in the human genome is surrounded by G4-prone sequences with peaks at 1.84 before p53 RE and long G4-prone sequence with the peak at 1.32 just after p53 RE and with a maximum peak with G4Hunter score 3.2).

p53 is a transcription factor that recognizes a 20-bp long DNA motif. However, chromatin immunoprecipitation has shown that many p53 targets do not contain a classical full-length p53 RE, but can be formed by half-site [21], or do not contain classical target sequences [39]. Non-canonical DNA motifs are transcriptionally active for wild type and mutant p53 proteins [40] and local DNA structures are important determinants for protein-DNA binding [41]. Recently, the interaction of p53 with G4s has been demonstrated [26]. Even if it was demonstrated that G4 structures are often located in gene regulatory sequences in the human genome [42] and there are many studies of p53 target genes [16,39], a combined study of both features is missing. Therefore, we performed additional analyses of 100 bp sequence surrounding the p53-target sequence in the PUMA gene promoter. Interestingly, there are several potential G4-prone sequences in close proximity to the PUMA p53-target sequence (Figure 7). The G4-prone sequence is located tightly before p53 RE (−33 to −1 before p53 RE, max. G4Hunter score in this area 1.84) and several G4-prone sequences are located after the p53 RE including a G4Hunter score of 1.32 immediately after the p53 RE—location 0–25—and another further downstream (starting either 21, 45, and 58 nucleotides after the p53 RE; highest G4Hunter score of 3.2 for the sequence: GGGGGCGGGG CGGGGCGGGG CGGGG, peak at 71 nucleotides after p53 RE).

**Figure 8:**
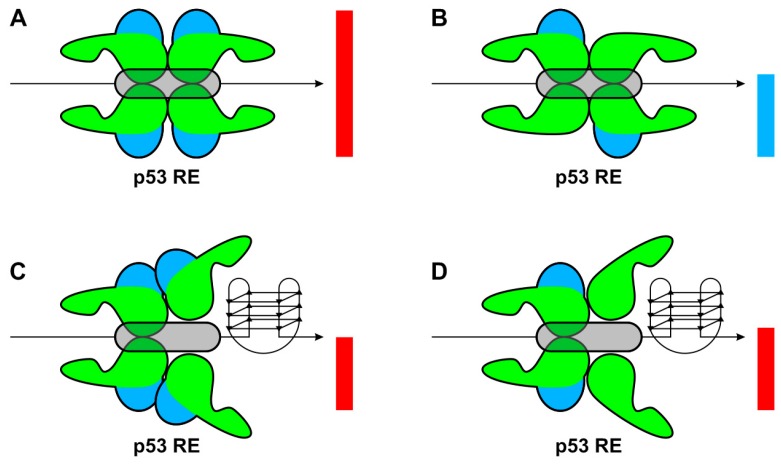
Schematic model of p53 isoforms binding to a RE associated with G4 sequence. (**A**) FLp53α or ∆40p53α bind effectively to the RE and there is a high or moderate level of transactivation. (**B**) ∆133p53α and ∆160p53α inhibit FLp53α transactivation, (**C**) the presence of a G4 close to the RE decreases accessibility of the TA domains and FLp53α transactivation, (**D**) which is not more inhibited by ∆133p53α and ∆160p53α, although steric protein orientation is impaired due to the G4 structure. TA is the blue domain, rest of the protein is in green, column represent transactivation induced by p53 complex (red column FLp53α, blue FLp53α with ∆133p53α and ∆160p53α isoforms).

Even though the localization of both p53 RE and G4 sequences have been shown in the genome, the roles of G-quadruplexes in regulating transcription by p53 isoforms have not been evaluated. Therefore, we prepared a model system and analyzed the impact of a sequence endowed with high propensity to adopt a G4 structure positioned either upstream or downstream of a moderately active p53 RE using yeast reporter strains. FLp53α protein and its ∆-isoforms failed to transactivate a minimal promoter when only a G4-prone sequence was inserted at the site. It has been shown recently that G4s have an inhibitory effect on translation in vivo in the yeast system [43]. Our results showed that ∆160p53α expressed together with FLp53α decreased transactivation at the p53 RE. These new data are in agreement with previously published apoptosis assays, where ∆160p53 inhibits apoptosis, in contrast to ∆133p53 [44]. On the other hand, the ∆133p53α and ∆160p53α isoforms failed to decrease transactivation of the p53 RE presented together with a G4-prone sequence in front of the RE; in fact, there was a slight increase in transactivation (Figure 5A). This result suggests that hetero-tetramerization of ∆133p53α or ∆160p53α with FLp53α (contrary to ∆40p53α) does not inhibit transactivation at p53 targets associated with a G4 structure, while in the case of ∆40p53α, competition between isoform specific homo-tetramers or the formation of hetero-tetramers can lead to the inhibition of the transactivation potential of FLp53α at these sites (Figure 8).

Therefore, it appears that the composition of the p53 isoforms could be a selective determinant in p53 transactivation specificity, resulting not only from the p53 RE sequence, but also from structural DNA features, particularly a G4 upstream or downstream of the p53 RE. The G4-prone sequences localized in close proximity to the PUMA p53 RE suggests that G4 formation could be an additional feature that determines the effectiveness of p53 transcriptional regulation. The co-expression of different p53 isoforms may increase plasticity through a compromise between effective FLp53 homotetramers at RE sites embedded in structurally favorable contexts and less effective, but sterically more beneficial heterotetramers, at RE sites flanked by structured motifs such as G4.

## 4. Methods

### 4.1. Preparation of Plasmids to Express p53α Isoforms

Vectors containing the coding sequences of p53α isoforms were prepared by the Gateway cloning system (detailed in [45]). As the destination vector, pAG414GALccdB-HA containing the inducible GAL promoter and pAG415GPDccdB-HA with the constitutive GPD promoter were used. Destination vectors containing the cDNAs of p53α isoforms were isolated from *E. coli* STBL3 strain using a commercial plasmid extraction kit (Omega-Biotek, Norcross, USA).

### 4.2. Preparation of Yeast Isogenic Strains by Delitto Perfetto Homologous Recombination

*S. cerevisiae* haploid strain yLFM-ICORE (MATα leu2–3nic strains, 112 trp1–1 his3–11,15 can1–100; ura3–1; ade2:RE:pCyc1::LUC1) was used for deriving a panel of isogenic reporter strains, which differ in the presence of a p53 RE and a G4 prone sequence (Table 1). The double counterselectable- REporter ICORE cassette was replaced by a targeting oligonucleotide, consisting of 30 nt flanking homology and the RE + G4 as an intervening sequence, following the protocol described in [46]. Replacement was facilitated by induction of a single site-specific DNA double strand break at the ICORE site by the homing endonuclease I-SceI, selected by exploiting resistance to 5-fluoro-orotic acid caused by loss of the ICORE cassette and confirmed by colony PCR and Sanger sequencing. The obtained yeast reporter strains differing in the p53 target site were purified and transformed with a plasmid for the expression of specific p53α isoforms.

### 4.3. Circular Dichroism (CD) Spectroscopy

CD measurements were carried out in a Jasco 815 (Jasco International Co. Ltd., Tokyo, Japan) dichrograph in 1 cm path-length quartz Hellma microcells placed in a thermostatically regulated cell holder at 23 °C. A set of four scans was averaged for each sample with a data pitch of 0.5 nm and 100 nm/min scan speed. CD signal was expressed as the difference in the molar absorption, ∆ε of the left- and right-handed circularly polarized light, molarity being related to DNA strands; buffer: 50 mM KCl, 5 mM Tris/HCl pH 8.

### 4.4. Transformation of Yeast Strains

Yeast were transformed by a method based on mixing cells and DNA in the presence of lithium acetate, TE, PEG, DMSO and performing heat shock, starting from saturated overnight cultures [47]. Double transformants were selected by auxotrophic selection on plates lacking both tryptophan and leucine.

### 4.5. Luciferase Assay

Purified transformant colonies were inoculated on 96-well plates in 120 µL selective media containing 2% raffinose as a carbon source and different concentrations of galactose to induce p53α isoform expression from the GAL promoter of the pAG414GAL vector. Luciferase was measured as described [40]. To ascertain p53 protein expression, samples used for the transcription analysis were also used to prepare protein extracts for immunodetection by western blotting.

### 4.6. Western Blot

Yeast cell lysis was performed as described [48]. Protein extracts were quantified using the Bradford assay. Proteins (80 µg) were electrophoresed using 12.5% acrylamide sodium dodecyl sulfate polyacrylamide gel electrophoresis (SDS-PAGE) and transferred to a nitrocellulose membrane. Specific antibodies directed against p53 were donated by Dr. Vojtěšek and the membranes were incubated as described [49,50,51]. The signal was detected using the ECL Select reagent (Pierce Fast Western Blot Kit, Thermo Fisher, WA, USA) and results were visualized as chemiluminiscence on LAS 3000. Results are shown in Figure S1.

### 4.7. Statistical Analysis

Transactivation data were plotted as fold luciferase induction relative to a control reporter activity, measured with cells that do not contain a p53 expression plasmid and cultured under the same conditions. Mean and standards deviation of at least three biological replicates are presented. Statistical significance was evaluated using Student’s t-test.

### 4.8. G4Hunter Analyses

The DNA sequence of the p53RE that regulates PUMA on chromosome 19 including 100 bp before and 20-bp after the p53RE was downloaded in FASTA format from the National Center for Biotechnology Information (NCBI) [52]. The sequence was analyzed by G4Hunter web [34] for the presence and localization of G-quadruplex prone sequences with parameters of “25” for window and G4Hunter score above 1.2.

## Figures and Tables

**Table 1:** Sequences cloned into luciferase promoter regions into yeast isogenic reporter strain (PUMA sequence – highlighted by grey, G-repeats – bold).

Region	Sequence 5’−3’
PUMA	CTGCAAGTCCTGACTTGTCC
PUMA–G4	CTGCAAGTCCTGACTTGTCC **GGGGCGGGGGACGGGGGAGGGG**
G4–PUMA	**GGGGCGGGGGACGGGGGAGGGG** CTGCAAGTCCTGACTTGTCC
G4	**GGGGCGGGGGACGGGGGAGGGG**

## Supplementary Materials

Supplementary materials can be found at https://www.mdpi.com/1422-0067/21/1/127/s1.
