# Supplementary material for: The Influence of Quadruplex Structure in Proximity to P53 Target Sequences on the Transactivation Potential of P53 Alpha Isoforms
Source: Int J Mol Sci. 2019 Dec 24;21(1):127. doi: 10.3390/ijms21010127 (PMC6982142; doi:10.3390/ijms21010127)
Supplement: Supplementary file 1 [file ijms-21-00127-s001.pdf]

The Influence of Quadruplex Structure in Proximity to P53 Target Sequences on the Transactivation Potential of P53 Alpha Isoforms

Otília Porubiaková, Natália Bohálová, Alberto Inga, Natália Vadovičová, Jan Coufal, Miroslav Fojta and Václav Brázda

The immunoblot shows the relative expression of FLp53α under the constitutive GPD promoter (lanes 2-5), confirms the inducibility of the GAL1 promoter (lanes 6 and 7), and establishes that FLp53α protein levels are not reduced in the double transformants with Δ40, Δ133, or Δ160 p53 expression plasmids. Unfortunately, we were unable to show the expression of p53 isoforms that lack the complete N-terminal. Ponceau S staining was used as control. Although the lack of antibodies that can detect with high affinities all p53 isoforms represents a limitation, the use of DO-1 allowed us to confirm FLp53α expression in yeast.

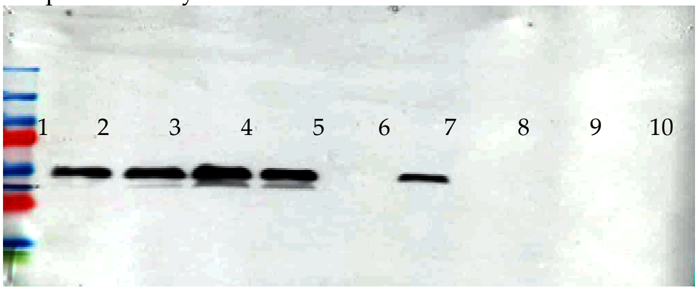

Figure S1a. Immunoblot analysis of single and double transformants in G4-PUMA strain. Immunoprecipitation of the p53alpha isoforms expressed under inducible GAL1 promoter (7 – FLp53αGAL in 2% galactose, 8 – Δ40p53α in 2% galactose, 9 – Δ133p53α in 2% galactose, 10 – Δ160p53α in 2% galactose) and coexpressed with FLp53alpha expressed under constitutive GPD promoter (2 – FLp53αGPD, 3 – FLp53αGPD + Δ40p53α, 4 – FLp53αGPD + Δ133p53α, 5 – FLp53αGPD + Δ160p53α) and control of FLp53alpha expressed under inducible GAL1 promoter in media without galactose (line 6). The samples were lysed in 2x RIPA lysis buffer with 1x ppi (inhibitor of proteases) and detected by DO-1 antibody. After SDS-PAGE and Western blotting the immunoprecipitated p53 proteins were visualized with mouse anti-mouse horseradish-peroxidase-conjugated secondary antibody. (Line 1 – Thermo Scientific™ PageRuler™ Plus Prestained 10-250kDa Protein Ladder.).

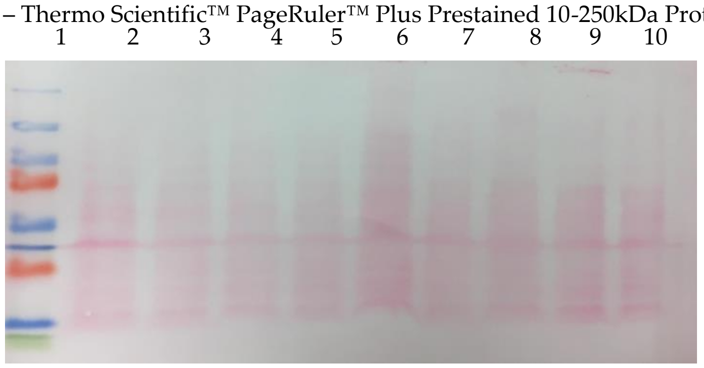

Figure S1b. Control of lysate loading and transfer by Ponceau S staining. Immunoprecipitation of the p53alpha isoforms expressed under inducible GAL1 promoter (7 – FLp53αGAL in 2% galactose, 8 – Δ40p53α in 2% galactose, 9 – Δ133p53α in 2% galactose, 10 – Δ160p53α in 2% galactose) and coexpressed with FLp53alpha expressed under constitutive GPD promoter(2 – FLp53αGPD, 3 – FLp53αGPD + Δ40p53α, 4 – FLp53αGPD + Δ133p53α, 5 – FLp53αGPD + Δ160p53α) and control of FLp53alpha expressed under inducible GAL1 promoter in media without galactose (line 6). (Line 1 – Thermo Scientific™ PageRuler™ Plus Prestained 10-250kDa Protein Ladder.).
